# *De novo* and inherited pathogenic variants in collagen‐related osteogenesis imperfecta

**DOI:** 10.1002/mgg3.559

**Published:** 2019-01-24

**Authors:** Lidiia Zhytnik, Katre Maasalu, Binh Ho Duy, Andrey Pashenko, Sergey Khmyzov, Ene Reimann, Ele Prans, Sulev Kõks, Aare Märtson

**Affiliations:** ^1^ Department of Traumatology and Orthopedics University of Tartu Tartu Estonia; ^2^ Clinic of Traumatology and Orthopedics Tartu University Hospital Tartu Estonia; ^3^ Hue University of Medicine and Pharmacy, Hue University Hue Vietnam; ^4^ Department of Pediatric Orthopedics Sytenko Institute of Spine and Joint Pathology, AMS Ukraine Kharkiv Ukraine; ^5^ Centre of Translational Medicine University of Tartu Tartu Estonia; ^6^ Department of Pathophysiology University of Tartu Tartu Estonia; ^7^ Centre for Comparative Genomics Murdoch University Perth Australia; ^8^ Perron Institute for Neurological and Translational Science University of Western Australia Perth Australia

**Keywords:** bone fragility, collagen, *de novo*, osteogenesis imperfecta, Sanger sequencing

## Abstract

**Background:**

Osteogenesis imperfecta (OI) is a rare genetic bone fragility disorder. In the current study, differences between the genotypes and phenotypes of *de novo* and inherited collagen‐related OI were investigated.

**Methods:**

A comparative analysis was performed of the genotypes and phenotypes of 146 unrelated inherited and *de novo* collagen I OI cases from Estonia, Ukraine, and Vietnam. Mutational analysis of the subjects and all available parents were performed with Sanger sequencing.

**Results:**

Results showed that 56.16% of the OI cases were caused by *de novo* pathogenic variants. The proportion of OI types OI1, OI4, and OI3 among subjects with inherited OI was 45.31%, 46.88%, and 7.81%, respectively. Among subjects with *de novo* OI, the proportions of OI types (OI1, OI4, and OI3) were almost equal. Both inherited and *de novo* OI pathogenic variants occurred more often in the *COL1A1* gene than in the *COL1A2*. The majority of *de novo* cases were missense pathogenic variants, whereas inherited OI was mostly caused by loss of function pathogenic variants.

**Conclusion:**

In summary, there were significant differences between the phenotypes and genotypes of subjects with *de novo* and inherited OI. These findings may promote the further understanding of OI etiology, and assist with diagnostics procedures, as well as with family planning.

## 1 INTRODUCTION

Osteogenesis imperfecta (OI) is a rare genetic disorder, characterized with congenital bone fragility. Prevalence of a disorder is estimated as 1/10–20,000 (Marini et al., 2017; Van Dijk & Sillence, 2014b). Primary feature of OI is susceptibility to bone fractures. Secondary features that may be present in individuals with OI are as follows: blue sclerae, hearing loss, dentinogenesis imperfecta (DI), joint hypermobility, and short stature (Byers & Steiner, 1992; Marini et al., 2017; Sillence, Senn, & Danks, 1979). OI is characterized by the variability of genotypes and phenotypes, ranging from mild osteopenia to perinatal lethality. Sillence OI types correspond to severity of the disorder as follows: type OI1—mild, type OI2—lethal, type OI3—severe, type OI4—moderate OI (Van Dijk & Sillence, 2014b).

Although there are 20 different recessive genes connected to OI pathology, about 70%–90% of patients with OI harbor dominant pathogenic variants in the *COL1A1* (OMIM accession number 120150) and *COL1A2* (OMIM accession number 120160) genes (Marini et al., 2017; Van Dijk & Sillence, 2014a; Womack, 2014). These genes code for collagen type I α1 and α2 chains, respectively. Collagen type I is an essential structural protein and is the most abundant structural protein in the human body (Gelse, Pöschl, & Aigner, 2003). Collagen chains consist of a Gly‐X‐Y triplet motifs were every third position is occupied with glycine. The importance of collagen type I is evidenced by its high conservation among vertebrates (Bonod‐Bidaud & Ruggiero, 2013; Gelse et al., 2003; Stover & Verrelli, 2011). Collagen type I naturally lacks variation on its termini, especially in the C‐terminal domain. These termini are highly conserved, as they have vital functions, such as collagen assembly, transport, and signaling (Stover & Verrelli, 2011).

Pathogenic variants in the *COL1A1/2* genes cause bone fragility, of varying severity. Loss of function (LoF) pathogenic variants in *COL1A1/2* genes correspond to haploinsufficiency and a collagen type I quantitative defect. Dominant negative pathogenic variants (i.e. missense substitutions) in the collagen type I structure correspond to a collagen type I qualitative defect (Ben Amor, Glorieux, & Rauch, 2011; Marini et al., 2007; Mendoza‐Londono et al., 2015; Shapiro, 2014). Gly substitution in the α1/2 chains usually correlate with more severe phenotypes. The severity of a phenotype caused by a dominant negative variant is also influenced by position of a substitution (Marini et al., 2007). Dominant negative pathogenic variants cause the synthesis of structurally abnormal collagen, which may cause endoplasmic reticulum stress and apoptosis of osteoblasts; missense pathogenic variants are therefore more deleterious than LoF pathogenic variants (Bonod‐Bidaud & Ruggiero, 2013; Lisse et al., 2008). Furthermore, dominant negative pathogenic variants appear more frequently, compared to LoF (i.e. frameshift and splice site) pathogenic variants, in sporadic genetic diseases (de Ligt, Veltman, & Vissers, 2013; Veltman & Brunner, 2012).

Being a rare hereditary disorder of connective tissue, OI has traditionally been described as a genetic disorder which runs in families. However, both inherited and *de novo* etiology of OI mutations exist. *de novo* pathogenic variants are sporadic variants appearing for the first time in a given family. These pathogenic variants are known to be more deleterious and to impose a greater load on a phenotype than inherited variants (Acuna‐Hidalgo, Veltman, & Hoischen, 2016; Veltman & Brunner, 2012). In the absence of reproductive selection, *de novo* pathogenic variants may cause congenital malformations, sporadic syndromes, and rare disorders to remain prevalent in a population (Veltman & Brunner, 2012). As noted by Acuna‐Hidalgo et al. (2016), *de novo* variants do not differ in prevalence across populations and are the main source of neurodegenerative and developmental disorders. With the availability of sequencing techniques, our understanding of the role of *de novo* pathogenic variants in both common and rare genetic disorders is changing, bringing with it increased knowledge about newly arising pathogenic variants.

The proportion of *de novo* OI mutations has previously been evaluated as being between 35%–60%, which is lower than that of other musculoskeletal disorders, such as achondroplasia (80%) (Ornitz & Marie, 2002; Osteogenesis Imperfecta | 978‐0‐12‐397165‐4 | Elsevier; Trotter & Hall, 2005).

Although many *de novo* OI cases have previously been described (Maasalu et al., 2015; Steiner, Adsit, & Basel, 1993; Tongkobpetch et al., 2017; Yin et al., 2018), there does not appear, to date, to have been any description or comparative analysis between *de novo* and inherited collagen I pathogenic variants. In the current study, data on 146 OI patients with either *de novo* or inherited *COL1A1/2* pathogenic variants were analyzed. Subjects with *de novo* or inherited *COL1A1/2* pathogenic variants were compared in terms of genotype characteristics and OI phenotype severity.

## 2 MATERIALS AND METHODS

### 2.1 Ethical compliance

The study was approved with the Ethical Review Committee on Human Research of the University of Tartu (Permit no. 221/M‐34), the ethical review board of Hue University Hospital (approval No. 75/CN‐BVYD) and the Sytenko Institute of Spine and Joint Pathology of the Ukrainian Academy of Medical Sciences.

### 2.2 Subjects

Study participants and data were from the OI database of the Clinic of Traumatology and Orthopedics, University of Tartu, Estonia for research purposes. The database includes 237 OI families, of Estonian, Ukrainian, and Vietnamese origin. Estonian OI patients from the register of Clinic of Traumatology and Orthopedics, Tartu University Hospital were enrolled into the database. As a result of collaboration between University of Tartu and Hue University of Medicine and Pharmacy, Ukrainian Association of Crystal People, Sytenko Institute of Spine and Joint Pathology, OI families from Ukraine and Vietnam attended an interview and clinical examination with researchers from the University of Tartu. Patients and their relatives were enrolled into OI database of the Clinic of Traumatology and Orthopedics, University of Tartu, Estonia. Patients with other skeletal disorders were excluded from the database. A comparative analysis of *de novo* and inherited OI cases, with OI due to a pathogenic variant in either *COL1A1* or *COL1A2*, was performed.

A total number of 146 unrelated subjects, diagnosed with OI (types OI1–OI4) and harboring *COL1A1/2* pathogenic variants participated in the study. Patients were classified as OI1–OI4, according to the observed clinical features. Clinical and genealogical data were recorded from the patients’ spoken words. Blood samples were obtained (for DNA analysis) from all available affected family members and their close healthy relatives. *COL1A1/2* mutational analysis was performed with Sanger sequencing. Detailed information has been reported in previous studies (Binh et al., 2017; Ho Duy et al., 2016; Zhytnik et al., 2017). In this current study, the cohort was comprised of Estonian (27), Vietnamese (57), and Ukrainian (62) OI patients and represented the youngest affected proband of every kindred. Of the cohort, 77 subjects were females and 69 were males. The age range of the subjects was from 2 months to 65 years of age.

### 2.3 Genealogical analysis and detection of *de novo* cases

Genealogical data were obtained in spoken from subjects during oral interviews and included OI family history; family consanguinity; history of miscarriages; parental ages; and health status (Binh et al., 2017; Zhytnik et al., 2017).

Based on OI family history, the inheritance pattern for every kindred was determined. Cases with multiple affected individuals across different generations were considered to be inherited OI. Cases without a previous OI history were considered to be *de novo*. The inheritance pattern of *de novo* pathogenic variants was identified on the basis of zygosity of the detected pathogenic variant, and later verified with the mutational analysis in parents. Pedigree trees were constructed using the “Kinship2” package in the R statistical program v3.3.2. (R team, Austria).

### 2.4 Controls

The absence of pathological variants harbored by subjects with *de novo* OI was checked for and confirmed in all available parents. The total number of parent‐child trios was 45. The total number of parent–child duos, that is where only one parent was available, was 29. No parent samples were available for eight of the subjects. In cases where parents’ DNA samples were not obtained, the *de novo* state of the disorder was defined on the basis of negative OI history in the family, and the heterozygous state of the OI‐causative pathogenic variant.

### 2.5 Mutational analysis of healthy parents

3 ml of an EDTA‐preserved whole blood sample of *de novo* probands’ healthy parents was used for genomic DNA purification, using the Gentra Puregene Blood Kit (Quiagen, Germany) in accordance with the manufacturer's protocol, and stored at −80°C.

In order to affirm the absence of an OI‐causative pathogenic variant in healthy parents, Sanger sequencing of an exon carrying a subject's pathogenic variant was performed. PCR amplification, Sanger sequencing, and analysis of the sequencing products were each performed as described in previous studies (Ho Duy et al., 2016; Zhytnik et al., 2017).

Sequence products were also aligned to the GenBank human reference genome sequences of *COL1A1* (gDNA NG_007400.1, complementary (cDNA) NM_000088.3) and *COL1A2* (gDNA NG_007405.1, cDNA NM_000089.3). The datasets used and analyzed during the study are available from the corresponding author upon reasonable request.

### 2.6 Statistical analysis

Associations between pathogenic variant nature, and genotype, OI type, and phenotype manifestations, were examined using Fisher's test for categorical variables. Averages of continuous variables were compared using the Student's *t*‐test. The threshold of statistical significance was a *p*‐value of <0.05. Statistical analysis of the data was completed with R v3.3.2. software (R Team, Austria) (Chen et al., 2012). All data analysis and laboratory operations were conducted at the University of Tartu, Estonia.

## 3 RESULTS

**Figure 1:**
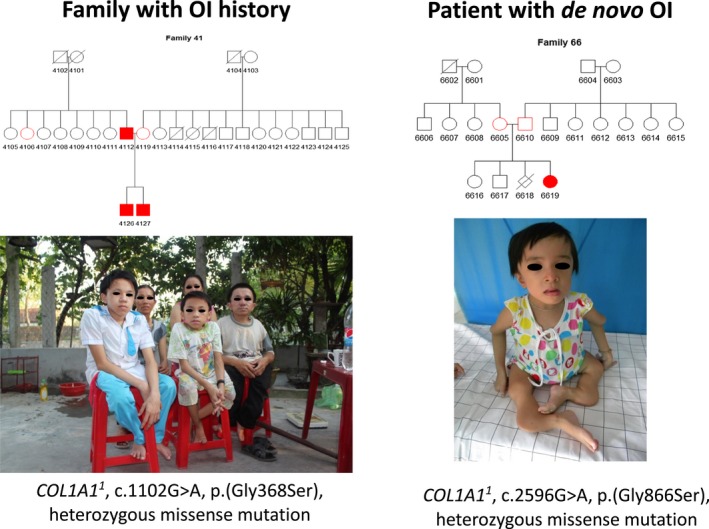
Pedigree trees, photographs, and genotypes of Vietnamese patients with familial (a) and *de novo* (b) osteogenesis imperfecta. ^1^ *COL1A1* GenBank reference sequence (gDNA NG_007400.1, cDNA NM_000088.3)

**Figure 2:**
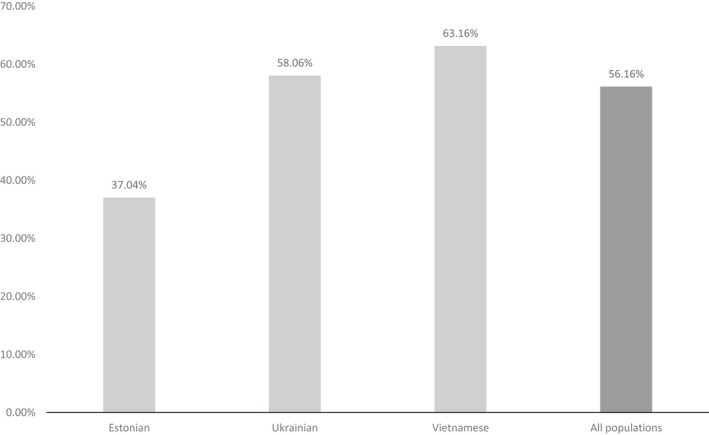
Proportion (%) of *de novo* mutations by population

Of the 146 OI cases analyzed, 82 (56.16%) subjects harbored *de novo* pathogenic variants. The proportion of *de novo* cases among Estonian (EE) OI subjects was 10/27 (37.04%); among Ukrainian (UA) 36/62 (58.06%); and among Vietnamese (VN) 36/57 (63.16%) (Figures 1 and 2; Table 1). Due to compound heterozygous pathogenic variants in seven patients, the total number of analyzed variants was 153. In *de novo* and inherited OI patient group, mean age was 14 and 15‐year‐olds, respectively.

**Table 1:** Characteristics of *de novo* and inherited OI cases

All patients	*de novo*	%	Inherited	%	Total	%	*p*‐value
Total	82	56.16	64	43.84	146	100	
EE	10	37.04	17	62.96	27	18.49	
UA	36	58.06	26	41.93	62	42.47	
VN	36	63.16	21	36.84	57	39.04	
Sex							
Males	34	49.28	35	50.72	69	47.26	0.1338
Females	48	62.34	29	37.66	77	52.74	
OI type							
OI1	22 (26.83%)	43.14	29 (45.31%)	56.86	51	34.93	**0.0002**
OI2	1 (1.22%)	100.00	0	0.00	1	0.69	
OI3	29 (35.36%)	85.29	5 (7.81%)	14.71	34	23.29	
OI4	30 (36.59%)	50.00	30 (46.88%)	50.00	60	41.09	
Fractures							
Number of total fractures (mean)	21.96		12.50		17.81		**0.0224**
Fractures per year (mean)	1.74		1.07		1.45		**0.0014**
Genotype							
*COL1A1* a	58 (65.91%)	52.25	53 (81.54%)	47.75	111	72.55	**0.0360**
*COL1A2* b	30 (34.09%)	71.43	12 (18.46%)	28.57	42	27.45	
Functional type							
Loss of function	28 (31.82%)	43.75	36 (55.38%)	56.25	64	41.83	**0.0063**
Nonsense and frameshift	12	37.50	20	62.50	32		
Splice site	16	50.00	16	50.00	32		
Missense	60 (68.18%)	67.42	29 (44.62%)	32.58	89	58.17	
Gly	48	68.57	22	28.57	70		**0.0139**
Gly‐Ser	24	61.54	15	38.46	39		0.5796
Architecture of mutations							
Transitions	54	56.84	41	43.16	95	62.09	0.6039
Transversions	24	63.16	14	36.84	38	24.84	
Indel	10	50.00	10	50.00	20	13.07	

Significant *p*‐values marked in bold.

a *COL1A1* GenBank reference sequence (gDNA NG_007400.1, cDNA NM_000088.3).

b *COL1A2* GenBank reference sequence (gDNA NG_007405.1, cDNA NM_000089.3).

### 3.1 Phenotypical signatures and OI manifestations in collagen I *de novo* pathogenic variants

**Figure 3:**
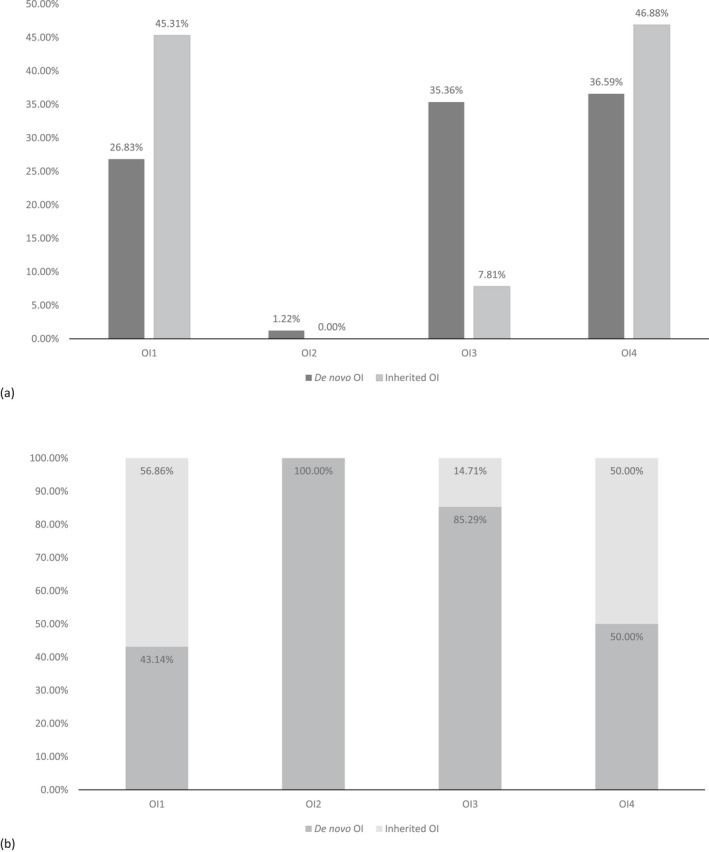
(a) Distribution of osteogenesis imperfecta (OI) types in collagen type I *de novo* and inherited OI. (b) Distribution of OI types in subjects with *de novo* or inherited OI

OI type was significantly associated with the proportion of *de novo* pathogenic variants (*p* = 0.0002) (Table 1). Among those subjects with *de novo* pathogenic variants, OI types OI1, OI3, OI4 were distributed almost evenly; although there was a slightly lower amount of type OI1 (26.83%) than of types 3 (35.36%) or OI4 (36.59%). In contrast, inherited OI was comprised of type OI1 in 45.31% of subjects, type OI3 in 7.81%, and type OI4 in 46.88%. Both type OI1 and type OI4 OI were proportionally more common in inherited OI cases than in *de novo* OI. Of the OI type III cases, 85.29% were *de novo* (Figure 3).

Tests on sclera color, hearing loss, and DI did not reveal any correlations with pathogenic variant nature (i.e. inherited or *de novo*). The majority of both *de novo* and inherited OI subjects had normal hearing and blue/gray eye sclera (80.49% and 84.38% for *de novo*; and 90.24% and 95.31% for inherited, respectively). The distribution of DI was almost equal for *de novo* (58.54%) and inherited (54.69%) OI subjects.

The number of total fractures (*p* = 0.0224), as well as fractures per year (*p* = 0.0014) were significantly higher in subjects with *de novo* pathogenic variants (means of 21.96 and 1.74, respectively) than in subjects with inherited OI (means of 12.50 and 1.07, respectively). However, when divided by types, differences in the number of fractures were only significant for fractures per year (mean 2.35, *p* = 0.0023) for subjects with *de novo* OI type 3, compared to patients with inherited OI type 3 (mean 0.95).

### 3.2 Proportion, functional type, and architecture of *de novo* and inherited *COL1A1/2* pathogenic variants

**Figure 4:**
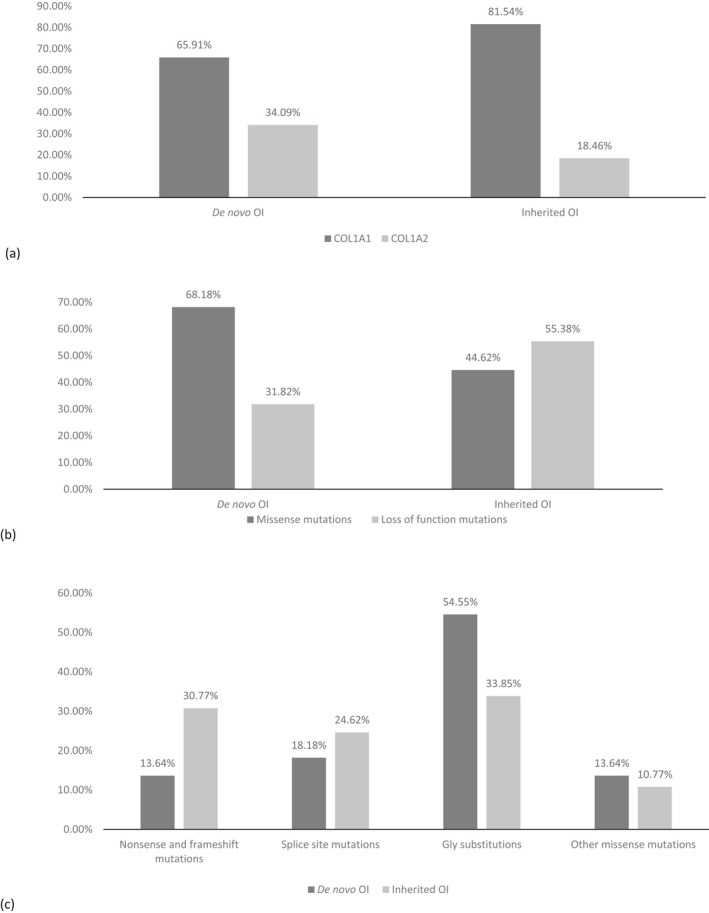
(a) Distribution of *COL1A1* ^*1*^ and *COL1A2* ^*2*^ mutations for *de novo* and inherited osteogenesis imperfecta (OI). (b) Functional types of mutations in *de novo* and inherited OI. (c) Collagen defect distributions in *de novo* and inherited OI. ^1^ *COL1A1* GenBank reference sequence (gDNA NG_007400.1, cDNA NM_000088.3). ^2^ *COL1A2* GenBank reference sequence (gDNA NG_007405.1, cDNA NM_000089.3)

A significant correlation was observed between the mutated gene and the nature of the pathogenic variant (*p* = 0.0360). Among *de novo* cases, *COL1A1* pathogenic variants comprised 65.91% (58/88), whereas among inherited cases, the proportion of *COL1A1* pathogenic variants was higher, at 81.54% (53/65) (Figure 4a; Table 1). Half of all identified *COL1A1* pathogenic variants were *de novo* (52.25%). The proportion of *COL1A2* variants was significantly higher in *de novo* (30/88, or 34.09%) than in inherited OI cases (12/65, or 18.46%) (Table 1, Figure 4a).

The proportion of *de novo* or inherited pathogenic variants was strongly correlated with the functional type of the defect (*p* = 0.0063). A higher proportion of missense changes was observed among subjects with *de novo* pathogenic variants (68.18%), whereas LoF variants were more common among inherited OI (55.38%) (Figure 4b). Of the *de novo* pathogenic variants, 54.55% were Gly substitutions (Figure 4c).

Of the 89 missense pathogenic variants, 60 (67.42%) were *de novo* in nature (Table 1). 48/70 (68.57%) of Gly substitutions occurred in *de novo* cases, as did 24/39 (61.54%) of Gly to Ser substitutions. Differences in the proportion of *de novo* and inherited Gly substitutions were significant (*p* = 0.0139), unlike Gly to Ser substitutions. The overall proportion of *de novo* LoF pathogenic variants in the studied cohort was 28/64 (43.75%), of which 16/32 (50.00%) were splice site pathogenic variants, and 12/32 (37.50%) were nonsense and frameshift pathogenic variants (Table 1).

The architecture of the pathogenic variants was also investigated. The majority of alterations were nucleotide transitions (95, or 62.09%), of which half were comprised of the most common G>A (50.66%) pathogenic variants. Transversions were observed 38 times (24.84%), of which 20 cases (13.07%) were with a spread G>T variant. Indels appeared 20 times; of which 14 were deletions (9.21%). None of these results showed significant differences between the *de novo* and inherited OI cases.

Overall, there was no significant correlation between the nature of a pathogenic variant (i.e. inherited or *de novo*) and its location. Variants were distributed evenly in the *COL1A1* gene. The majority of pathogenic variants altered the α1 chain region. Interestingly, pathogenic variants of the *COL1A2* gene only started from the exon 16 and altered the α2 chain domain.

## 4 DISCUSSION

The analyses revealed a high proportion of *de novo* pathogenic variants in the cohort of OI subjects. These results correlate with those of Steiner et al. (1993) and lend support to the idea that the majority (up to 60%) of OI cases arise as *de novo* pathogenic variants (Steiner et al., 1993). Interestingly, the lowest proportion of *de novo* cases was observed in the Estonian OI population. In the authors’ view, this population difference may be connected to the milder OI phenotypes of the Estonian subjects, which, compared to more severe OI phenotypes, have less impact on fitness and do not obstruct offspring production.

Osteogenesis imperfecta is a heterogenic skeletal pathology with incomplete penetrance and variable severity; the proportion of *de novo* pathogenic variants therefore differs according to OI type. Results showed that, among OI3 cases, 85.29% of pathogenic variants were *de novo*; this accords well with figures for achondroplasia, but was lower than previously reported rates of OI type 3 (Ornitz & Marie, 2002; Osteogenesis Imperfecta | 978‐0‐12‐397165‐4 | Elsevier; Trotter & Hall, 2005). This difference may be attributable to sample sizes, cohorts’ phenotype characteristics, and/or high inter‐ and intrafamilial variability of OI phenotypes. Consequently, the same pathogenic variant may cause OI of different severities, affecting the relative fitness of the harboring individuals. Considering the importance of the current topic to family planning and routine genetic testing, the additional factors which may shape OI phenotype warrant further investigation.

### 4.1 OI phenotype and etiology of the OI pathogenic variant

The most striking result to emerge from the data was that types OI1, OI4, and OI3 were distributed among *de novo* cases almost equally (26.83%, 36.59%, and 35.36%, respectively). Relatively lower proportions of types OI1 and OI4, or a relatively higher proportion of type OI3 were not observed among subjects with *de novo* pathogenic variants. A possible explanation for this result may be that severe sporadic (i.e. *de novo*) variants altering *COL1A1/2* genes may generate pathologically extreme, lethal phenotypes, which are mostly eliminated during early pregnancy; they were therefore not captured in this study. Consequently, the various nonlethal sporadic OI types arose with relatively similar frequency. More pronounced differences in the prevalence of the different OI types were observed in the inherited OI cases.

Furthermore, the relative proportions of the milder OI forms (i.e. types OI1 and OI4) were lower in *de novo* cases than in inherited cases. It can therefore be hypothesized that patients with mild *de novo* OI are under‐ or misdiagnosed, due to a negative OI family history and the variability of clinical characteristics. For example, mild *de novo* OI forms may be misinterpreted as child abuse or assumed to be other skeletal dysfunctions.

### 4.2 Genotype association with OI pathogenic variants’ etiology

As outlined earlier, the majority of collagen type I OI pathogenic variants alter the *COL1A1* gene. Compared to *de novo* pathogenic variants, the number of inherited *COL1A2* pathogenic variants is significantly lower. Some of the *COL1A2* gene alterations do not have pathogenic significance and may therefore stay undiagnosed. These same factors may play a role in the understanding of the lower number of *COL1A2* pathogenic variants among subjects with inherited OI. For example, previous generations may have been aware of light bone fragility in the family and thus did not connect it with any particular bone condition. They may also have lacked diagnostics compared to patients with *de novo* OI.

As noted above, a clear distinction was observed in the correlations between pathogenic variant type and pathogenic variant nature. More than half of inherited OI cases harbored LoF (or null‐allele) pathogenic variants, which generally lead to milder forms of OI. Interestingly, despite the fact that OI types were distributed almost equally among *de novo* OI, the majority of *de novo* pathogenic variants were structural. Consequently, the higher prevalence of LoF pathogenic variants among inherited OI cases may have been due to the higher fitness of mild OI cases.

The results from the analysis of mutational architecture also aligned with those of previous studies (Acuna‐Hidalgo et al., 2016; de Ligt et al., 2013; Stover & Verrelli, 2011). The majority of nucleotide changes in this study were G>A transitions, altering the chain domain of a protein. Fewer pathogenic variants altered termini regions of collagen type I chains, compared to the chain domain. At the same time, the higher overall prevalence of transitions compared to transversions may be explained by the instability of methylated CpG dinucleotides (CG sites) (Acuna‐Hidalgo et al., 2016). Regions rich in CG nucleotides are known to be highly expressive. Thus, most of the disease‐causing pathogenic variants, including OI, happen in coding regions, which are rich in CpG islands.

The implications of *de novo* pathogenic variants in musculoskeletal disorders is a vital issue for future research, which may advance our understanding of the disorders’ nature and etiology; highlight risks for both rare and common bone diseases; promote diagnostic approaches; and assist with family genetic counseling.

## 5 CONCLUSIONS

The present study analyzed the genotypes and phenotypes of *de novo* and inherited OI cases. Of these, the percentage of *de novo* OI cases was 56.16%. The majority of pathogenic variants were found to alter the *COL1A1* gene. *de novo* OI cases were characterized by a high prevalence of collagen qualitative defects, whereas inherited OI cases were characterized by quantitative collagen defects. As found in previous studies, mild *de novo* OI appeared to be under‐ or misdiagnosed. Further investigation should be focused on the issue of mild OI. The proportions of mild and moderate *de novo* OI cases were not lower than the proportion of severe *de novo* cases. The proportion of *COL1A2* variants among subjects with inherited OI was significantly lower than for subjects with *de novo* OI.

These results suggest that there are substantial differences between the genotype and phenotype characteristics of *de novo* and inherited OI. The authors believe that more attention should be given to OI diagnostics, medical awareness, and therapy development, as the disorder's prevalence may increase as a result of the numerous *de novo* cases.

The present study only investigated pathogenic variants of the *COL1A1* and *COL1A2* genes. However, with access to next‐generation sequencing, the further evaluation of overall differences in mutational load between inherited and *de novo* OI patients, and the contribution of these to complex phenotype variations, should shed additional light on etiology, pathological mechanisms, and the phenotype development of OI.

## CONFLICT OF INTERESTS

None declared.

## DATA SHARING

The datasets used and analyzed during the current study are available from the corresponding author on reasonable request.

## PATIENT CONSENT

Informed written consent from all subjects and controls, or their legal representatives, were collected prior to participation in the study.

## ETHICAL APPROVAL

In accordance with the Helsinki Declaration, the Ethical Review Committee on Human Research of the University of Tartu (Permit no. 221/M‐34), the ethical review board of Hue University Hospital (approval No. 75/CN‐BVYD) and the Sytenko Institute of Spine and Joint Pathology of the Ukrainian Academy of Medical Sciences have each authorized this ongoing study.
